# A case report of extremely rare case of fishbone penetration from stomach into spleen, causing splenic abscess, managed by spleen preserving surgery

**DOI:** 10.1016/j.ijscr.2024.110098

**Published:** 2024-07-30

**Authors:** Yasir Adam Fadlalla, Srinivasa Swamy Bandaru

**Affiliations:** General Surgery, Saqr Hospital, Ras Al Khaimah, Emirates Health Services, United Arab Emirates; Surgery, Ras AL Khaimah Medical and Health Sciences University, United Arab Emirates

**Keywords:** Splenic, Abscess, Stomach, Fishbone, Penetration, Peritonitis

## Abstract

**Introduction:**

Fishbone penetrating from the stomach into the spleen, causing a splenic abscess, is an extremely rare condition.

**Case presentation:**

We report a case of fishbone penetration from the stomach into the spleen, presenting as a splenic abscess and acute peritonitis, diagnosed pre-operatively with a contrast-enhanced CT scan of the abdomen and subsequently managed with spleen-preserving surgery.

**Discussion:**

Fishbone penetration from the stomach into the spleen, causing a splenic abscess, which is an extremely rare occurrence. We successfully diagnosed and managed this case with spleen-preserving surgery, and the patient recovered well.

**Conclusion:**

A rare case of fishbone penetration from the stomach into the spleen causing a splenic abscess was diagnosed radiologically pre-operatively and managed by a spleen-preserving procedure.

## Highlights

• Fishbone penetration from stomach into spleen
• Fishbone & splenic abscess, presenting as generalized peritonitis
• Managed by spleen preserving surgery
• Extremely rare case to be reported

## 1 Introduction and importance

Fishbone penetration from the stomach into the spleen, causing a splenic abscess, is an extremely rare condition presenting as an acute abdominal condition and comes with diagnostic difficulties and management. We report a case of this condition, which we diagnosed preoperatively and managed successfully with a spleen-conserving procedure. The work has been reported in line with the SCARE criteria [10]*.*

## 2 Case presentation

Clinical History: A 43-year-old male presented with severe abdominal pain of one-day duration. The pain started in the central abdomen and spread all over the abdomen. No additional abdominal symptoms or fever were present, and there was no previous surgery, chronic illnesses, or allergies. The review of systems was unremarkable, including psychiatry problems.

On clinical examination**:** Vital signs: tachycardia with a heart rate of 104/min, normal blood pressure and oxygen saturation**,** alert, conscious and oriented. Chest was clear with bilateral good air entry.

Abdomen showed generalized tenderness with rigidity and guarding suggesting acute peritonitis, normal hernial orifices, and external genitalia.

Laboratory investigation revealed a white blood cell count of 14,120, a neutrophil count of 78 %, a normal hemoglobin and platelet count, and a C-reactive protein of 35.7.

Renal and liver function tests were within normal limits, and serum amylase and lipase were normal.

**Fig. 1:**
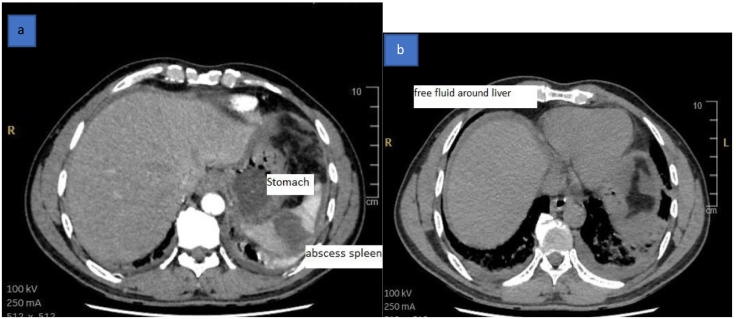
CT scan of the abdomen showing a: splenic abscess, b: free fluid around the liver.

**Fig. 2:**
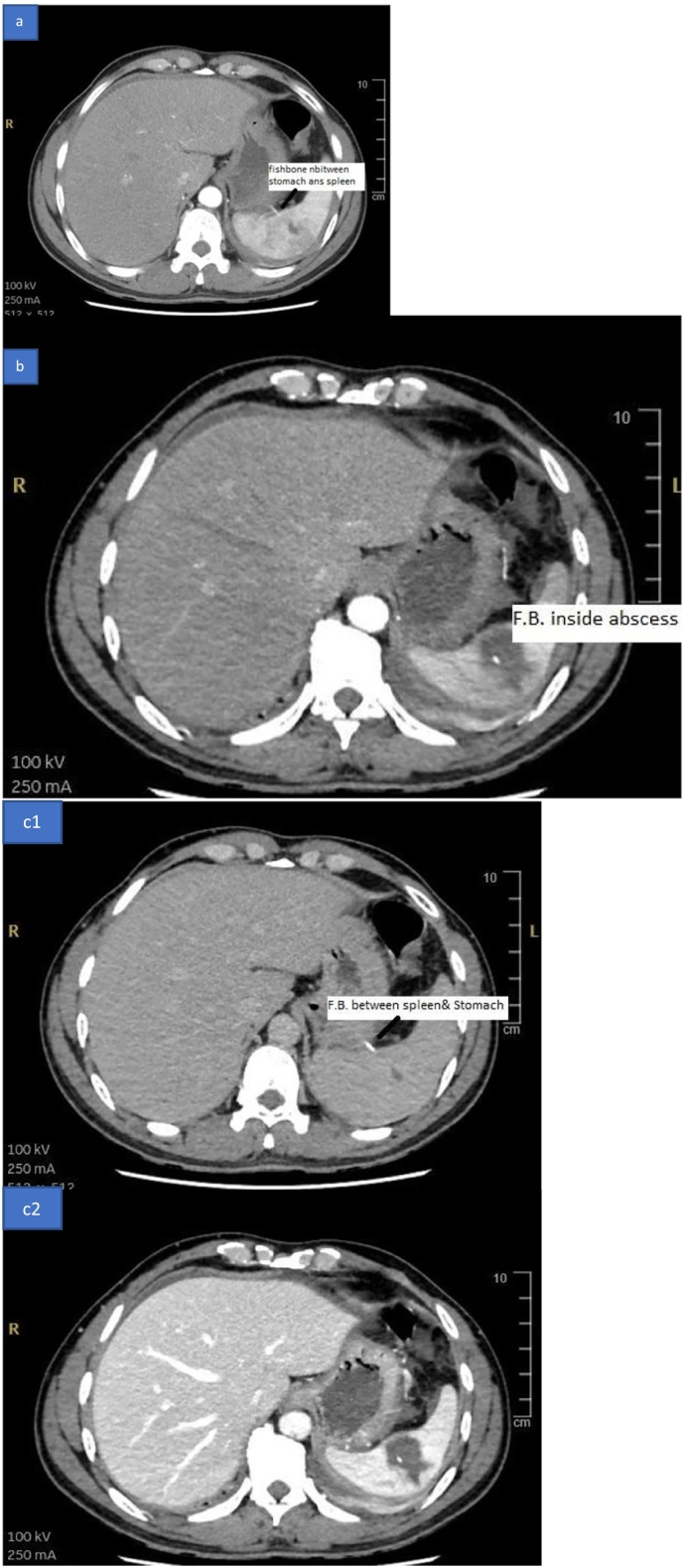
CT scan of the abdomen showing a & c1: Foreign body between stomach and spleen, b & c2: foreign body inside splenic abscess.

**Fig. 3:**
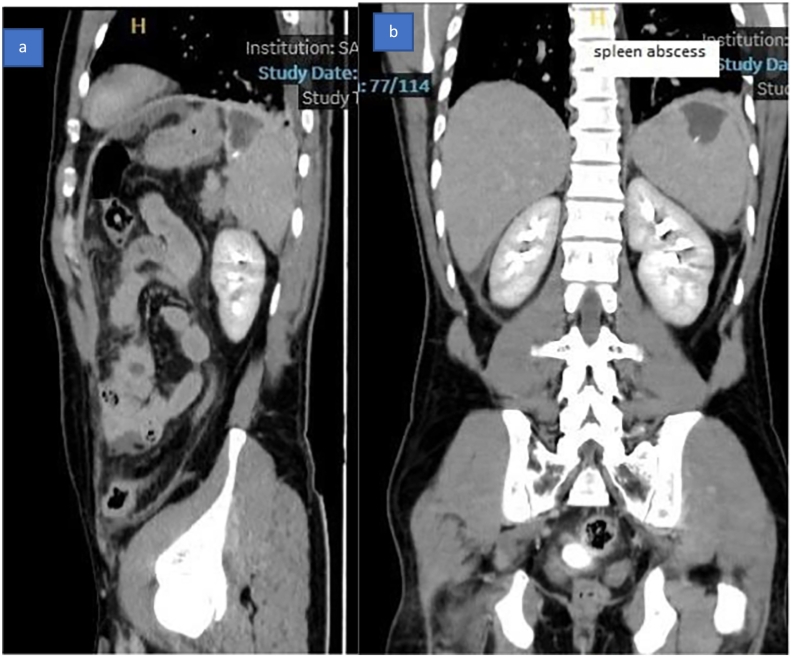
CT scan of the abdomen showing foreign body inside splenic abscess a: Sagittal view, b: Coronal view.

Radiological investigation: contrast-enhanced Computerized tomography scan of the abdomen: report as follows: Hypodense, non-enhancing focal area is seen within the spleen, measuring 3.8 × 3 × 2.4 cm *(*Fig. 1a and b*). A hyperdense* rod is seen extending from the center of the splenic hypodensity, extending medially through the splenic hilum, abutting the greater curvature of the stomach with no intragastric extension *(*Fig. 2a, b, c1, and c2). Mild thickening of jejunal loops Mesenteric fat stranding. Mild peri-splenic, perihepatic *(*Fig. 1a and b*),* and pelvic free fluid. Mild bilateral pleural effusion *(*Fig. 3a and b*)* and lower lung lobes ground glass densities and left small consolidation.

Management: Patient was admitted to the ward and started on intravenous ceftriaxone and metronidazole.

**Fig. 4:**
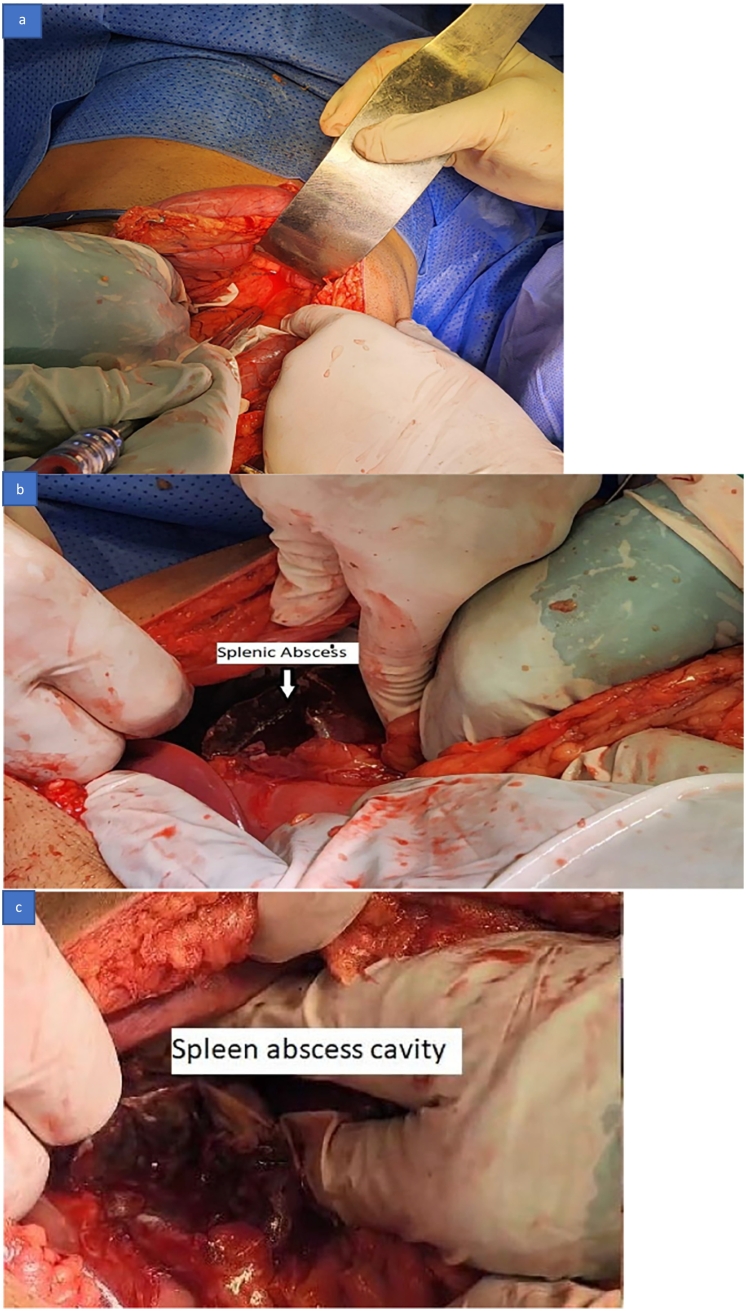
Surgery pictures showing a: splenic abscess, b & c: opened splenic abscess.

**Fig. 5:**
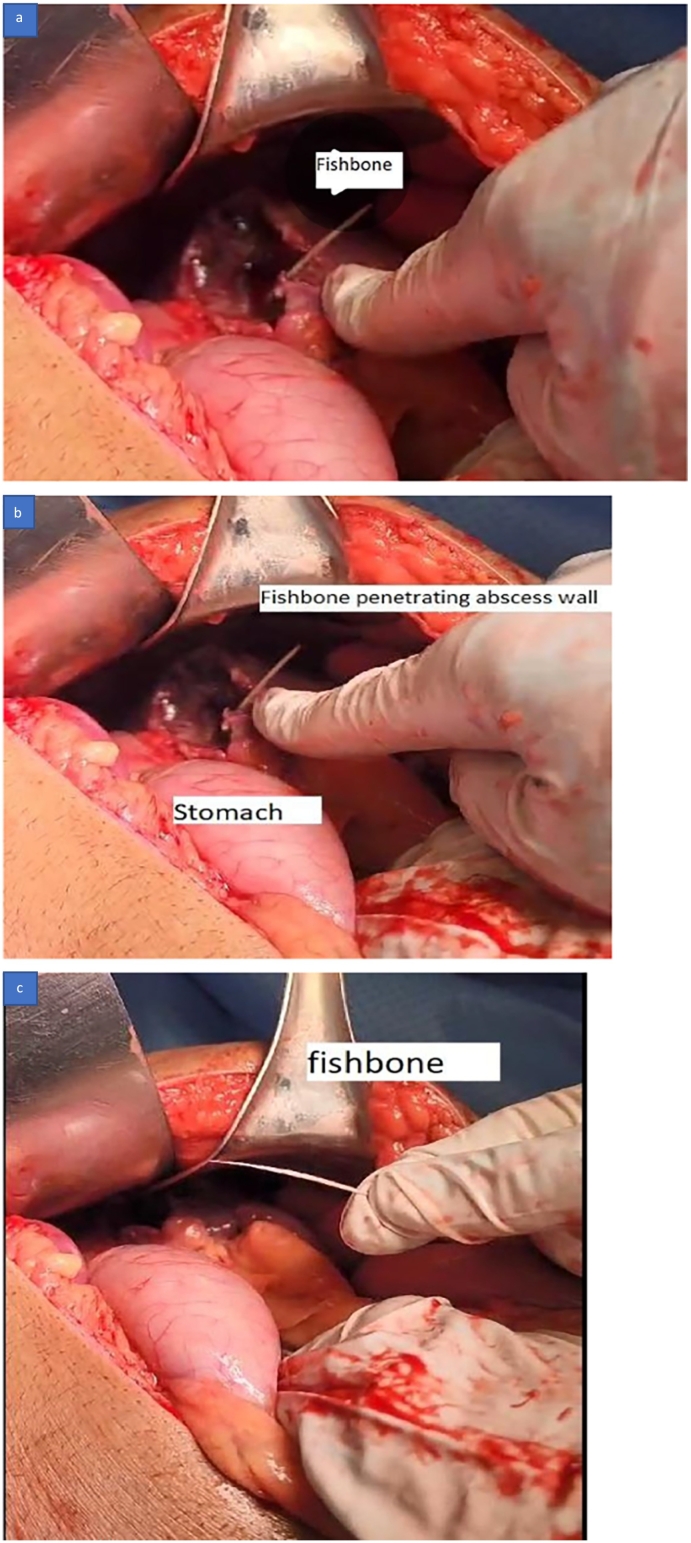
Surgery pictures showing a & b: Fishbone penetrating the splenic abscess wall, c: fishbone removed.

A plan was made for an emergency diagnostic Laparoscopy, possible laparotomy, and splenectomy. An informed patient consent was obtained. The patient underwent a diagnostic laparoscopy on the same day of admission, and the laparoscopy revealed an omentum covering the left hypochondrium with the finding of a splenic abscess at its upper pole and a fishbone communicating from the abscess to the lateral wall of the stomach with pus around 600 mL all over the abdomen. The decision was made to convert to open laparotomy, and the laparoscopy finding of a splenic abscess was confirmed *(*Fig. 4a, b, and c*).* The pus was suctioned, a sample was collected for culture sensitivity, and the omentum was separated cautiously from the superior pole of the spleen. Fishbone was found communicating from the outer wall of the stomach and going into the splenic abscess *(*Fig. 5a, b, and c*)*. Fishbone was removed (Fig. 5c*)*, deroofing of the splenic abscess was done (Fig. 4*c),* and an omentum plug was inserted into the splenic abscess cavity. Stomach integrity was checked by injecting methylene blue into the stomach through a nasogastric tube, and there was no leak of methylene blue dye. Saline lavage of the peritoneal cavity is done, a drain no. 18 is kept at the left upper abdomen, and a drain no. 18 is kept in the pelvis. Abdominal wall closure with no. 1 nylon loop and stapler applied to the skin.

Post-operative care and recovery: post-operatively, the patient recovered uneventfully. Inflammatory markers came down gradually. Both abdominal drains were removed on the 5th postoperative day, when their output became minimal. The patient tolerated a full diet by postoperative day three and was continued on intravenous antibiotics postoperatively until the 7th postoperative day. The patient was discharged home in good condition on the 7th postoperative day with a well-healing laparotomy wound. Pus for culture sensitivity showed no growth. The patient followed up at 2 weeks post-discharge and was doing well with no abdominal symptoms or fever.

## 3 Clinical discussion

After an initial clinical and radiological evaluation, the patient had surgery and a long, sharp fishbone was found going from the stomach to the spleen. This indicates that the splenic abscess was caused by a fishbone piercing through from the stomach. During surgery, it was discovered that there were no leaks at the point where the stomach was penetrated, indicating that the spot on the stomach wall where the object entered the spleen has since healed. The spleen-saving surgery was performed, and the patient had a smooth recovery before being discharged. The patient usually consumed fish, but couldn't remember the specific time of his last fish meal prior to the incident.

In a study, it was found that 83 % of reported foreign body perforations occurred in the ileocecal region, with rectosigmoid colon following closely behind [2]*.* These perforation sites are usually associated with peritonitis and abscess development [3]. Rarely, fishbone bowel perforation is complicated by the formation of liver abscesses [4]*.*

One case report literature review [6], it was noted that there has been a total of fifty-two reported cases of liver abscess due to fishbone migration from the digestive tract, with 25 cases showing clear perforation in the stomach and duodenum, and 27 cases where the perforation site was not determined [6]*.* Another instance was documented involving a liver abscess caused by a fishbone puncture originating from Meckel's diverticulum [7]*.* One case of porta hepatis abscess formation along with portal vein thrombosis was reported due to fishbone perforation with no obvious exit wound on the stomach [5]. A case of peripancreatic head of pancreas abscess reported due to sharp ingested bone perforation from the stomach was reported in the journal of internal medicine 2012 [8]. Some instances of objects moving from the duodenum to the right kidney were also recorded in a small number of cases [9]*.*

Our search in PubMed Central, google scholar and Clinical Key, using keywords related to splenic abscess caused by foreign bodies perforating from the stomach or bowel, did not uncover any case studies on this topic. Yet, a case study in the World Journal of Surgery [1] detailed a situation in which intraperitoneal bleeding occurred due to a fishbone puncturing the spleen, ultimately requiring splenectomy for treatment. The case we reported is fishbone perforation from the stomach into the spleen, causing a splenic abscess, diagnosed radiologically before surgery, and managed with spleen conserving surgery.

## 4 Conclusion

Fishbone penetration from the stomach into the spleen leading to the formation of a splenic abscess is an extremely rare condition that presents as a case of an acute abdomen with signs of peritonitis, diagnosed by a contrast-enhanced computerized tomography scan of the abdomen, and managed appropriately by timely surgical intervention with a spleen-preserving procedure.

## Abbreviations

mL: milliliter
CT: computerized Tomography
FRCS: fellow of royal college of Surgeons
M.S.: Master of Surgery

## Consent

Informed consent taken from patient and a copy of the consent is available for review by the editor in chief of this journal on request. No Patient Identifiers used in the case report.

## Ethical approval

Ministry of Health and Prevention, Research and Ethical Committee/RAK subcommittee, Saqr Hospital, Ras Al Khaimah, United Arab Emirates, approval reference number: MOHAP/REC/2024/52-2024-F-M.

## Funding

No funding is done by any entity.

## Author contribution

Both authors only have contributed for this case report, reviewed and approved by both.

## Guarantor

Dr. Yasir Adam Fadlalla is the guarantor for this Case report.

## Research registration number

A case report, not applicable.

## Conflict of interest statement

No conflict of interest from both authors.
